# 2-Methyl-1-phenyl­sulfonyl-1*H*-indole-3-carbaldehyde

**DOI:** 10.1107/S1600536811035665

**Published:** 2011-09-14

**Authors:** C. Ramathilagam, V. Saravanan, A. K. Mohanakrishnan, P. R. Umarani, V. Manivannan

**Affiliations:** a Department of Physics, AMET University, Kanathur, Chennai 603 112, India; b Department of Organic Chemistry, University of Madras, Guindy Campus, Chennai 600 025, India; c Department of Physics, Presidency College (Autonomous), Chennai 600 005, India; d Department of Research and Development, PRIST University, Vallam, Thanjavur 613 403, Tamil Nadu, India

## Abstract

In the title compound, C_16_H_13_NO_3_S, the sulfonyl-bound phenyl ring forms a dihedral angle of 84.17 (6)° with the indole ring system. An intra­molecular C—H⋯O hydrogen bond generates an *S*(6) ring motif. The crystal structure exhibits weak inter­molecular C—H⋯O hydrogen bonds and π–π inter­actions between the five- and six-membered rings of the indole group [centroid–centroid distance = 3.6871 (9) Å].

## Related literature

For the biological activities of indole compounds, see: Chai *et al.* (2006 ▶); Singh *et al.* (2000 ▶); Andreani *et al.* (2001 ▶). For related structures, see: Chakkaravarthi *et al.* (2007 ▶, 2008 ▶); Ramathilagam *et al.* (2011 ▶). For graph-set notation, see: Bernstein *et al.* (1995 ▶). 
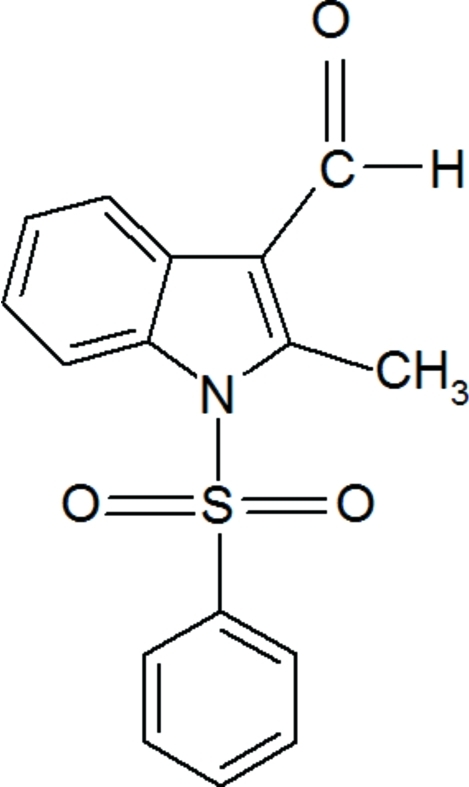


## Experimental

### 

#### Crystal data

C_16_H_13_NO_3_S
*M* *_r_* = 299.33
Monoclinic, 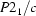

*a* = 11.6305 (5) Å
*b* = 8.4039 (4) Å
*c* = 14.3128 (8) Å
β = 93.126 (1)°
*V* = 1396.87 (12) Å^3^
*Z* = 4
Mo *K*α radiation
μ = 0.24 mm^−1^
*T* = 295 K
0.22 × 0.20 × 0.18 mm

#### Data collection

Bruker Kappa APEXII CCD diffractometer
Absorption correction: multi-scan (*SADABS*; Sheldrick, 1996 ▶) *T*_min_ = 0.949, *T*_max_ = 0.958
18442 measured reflections
4242 independent reflections
3212 reflections with *I* > 2σ(*I*)
*R*_int_ = 0.024

#### Refinement

*R*[*F* ^2^ > 2σ(*F* ^2^)] = 0.041
*wR*(*F* ^2^) = 0.116
*S* = 1.06
4242 reflections
191 parameters
H-atom parameters constrained
Δρ_max_ = 0.31 e Å^−3^
Δρ_min_ = −0.27 e Å^−3^

### 

Data collection: *APEX2* (Bruker, 2004 ▶); cell refinement: *SAINT* (Bruker, 2004 ▶); data reduction: *SAINT*; program(s) used to solve structure: *SHELXS97* (Sheldrick, 2008 ▶); program(s) used to refine structure: *SHELXL97* (Sheldrick, 2008 ▶); molecular graphics: *PLATON* (Spek, 2009 ▶); software used to prepare material for publication: *SHELXL97*.

## Supplementary Material

Crystal structure: contains datablock(s) global, I. DOI: 10.1107/S1600536811035665/ci5199sup1.cif

Structure factors: contains datablock(s) I. DOI: 10.1107/S1600536811035665/ci5199Isup2.hkl

Supplementary material file. DOI: 10.1107/S1600536811035665/ci5199Isup3.cml

Additional supplementary materials:  crystallographic information; 3D view; checkCIF report

## Figures and Tables

**Table 1:** Hydrogen-bond geometry (Å, °)

*D*—H⋯*A*	*D*—H	H⋯*A*	*D*⋯*A*	*D*—H⋯*A*
C5—H5⋯O1	0.93	2.28	2.8723 (19)	121
C12—H12⋯O3^i^	0.93	2.48	3.1664 (19)	131
C16—H16⋯O2^ii^	0.93	2.48	3.388 (2)	167

Symmetry codes: (i) 
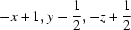
; (ii) 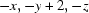.

**Fig. 1.**
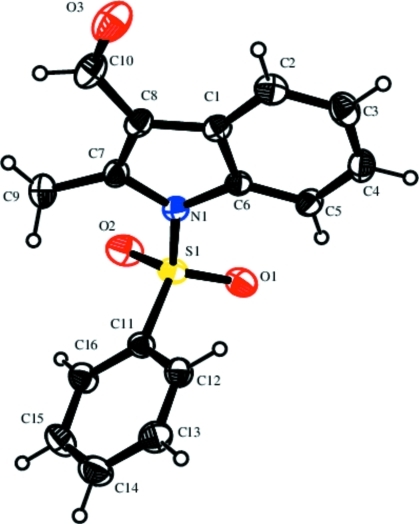
The molecular structure of the title compound, with atom labels and 30% probability displacement ellipsoids for non-H atoms.

## supplementary crystallographic information

### Comment

Indole derivatives are found in many natural products and these derivatives exhibit antibacterial, antifungal (Singh *et al.*, 2000) and antitumour activities (Andreani *et al.*, 2001). In addition, certain indole derivatives exhibit anti-hepatitis B virus (Chai *et al.*, 2006) activity.

The geometric parameters of the title molecule (Fig. 1) agree well with those observed in related structures (Chakkaravarthi *et al.*, 2007, 2008; Ramathilagam *et al.*, 2011). The dihedral angle between the benzene (C1–C6) and phenyl rings (C11–C16) is 83.81 (7)°. The sum of bond angles around N1 [359.9°] indicates *sp^2^* hybridization.

The molecular structure is stabilized by a weak intramolecular C—H···O hydrogen bond and the crystal packing is stabilized by weak intermolecular C—H···O hydrogen bonds. The intramolecular C5—H5···O1 hydrogen bond generates an S(6) ring (Bernstein *et al.*, 1995).

### Experimental

2-Methylindole-3-carboxaldehyde (5 g, 31.4 mmol) was dissolved in distilled benzene (100 ml). To this benzenesulfonylchloride (6.6 g, 4.8 ml, 37.7 mmol) and 60% aqueous NAOH (32g in 53ml) were added along with tetrabutyl ammonium hydrogensulfate (1.0 g). This two phase system was stirred at room temperature for 2h. It was then diluted with water (200 ml) and the organic layer was separated. The aqueous layer was extracted with benzene (2× 30 ml) and the combined organic extracts were dried (Na_2_SO_4_). The solvent was removed completely and the crude product was recrystallized from methanol (m.p 431–433 K).

### Refinement

H atoms were positioned geometrically and refined using riding model, with d(C–H) = 0.93 Å and *U*_iso_(H) = 1.2U_eq_(C) for aromatic C–H and d(C–H) = 0.96 Å and *U*_iso_(H) = 1.5U_eq_(C) for methyl C–H.

### Crystal data

**Table:**

C_16_H_13_NO_3_S	*F*(000) = 624
*M**_r_* = 299.33	*D*_x_ = 1.423 Mg m^−^^3^
Monoclinic, *P*2_1_/*c*	Mo *K*α radiation, λ = 0.71073 Å
Hall symbol: -P 2Ybc	Cell parameters from 4242 reflections
*a* = 11.6305 (5) Å	θ = 2.8–30.5°
*b* = 8.4039 (4) Å	µ = 0.24 mm^−^^1^
*c* = 14.3128 (8) Å	*T* = 295 K
β = 93.126 (1)°	Block, colourless
*V* = 1396.87 (12) Å^3^	0.22 × 0.20 × 0.18 mm
*Z* = 4	

### Data collection

**Table:**

Bruker Kappa APEXII CCD diffractometer	4242 independent reflections
Radiation source: fine-focus sealed tube	3212 reflections with *I* > 2σ(*I*)
graphite	*R*_int_ = 0.024
ω and φ scans	θ_max_ = 30.5°, θ_min_ = 2.8°
Absorption correction: multi-scan (*SADABS*; Sheldrick, 1996)	*h* = −8→16
*T*_min_ = 0.949, *T*_max_ = 0.958	*k* = −11→11
18442 measured reflections	*l* = −20→20

### Refinement

**Table:**

Refinement on *F*^2^	Primary atom site location: structure-invariant direct methods
Least-squares matrix: full	Secondary atom site location: difference Fourier map
*R*[*F*^2^ > 2σ(*F*^2^)] = 0.041	Hydrogen site location: inferred from neighbouring sites
*wR*(*F*^2^) = 0.116	H-atom parameters constrained
*S* = 1.06	*w* = 1/[σ^2^(*F*_o_^2^) + (0.051*P*)^2^ + 0.3293*P*] where *P* = (*F*_o_^2^ + 2*F*_c_^2^)/3
4242 reflections	(Δ/σ)_max_ = 0.001
191 parameters	Δρ_max_ = 0.31 e Å^−^^3^
0 restraints	Δρ_min_ = −0.27 e Å^−^^3^

### Special details

**Table:**

Geometry. All e.s.d.'s (except the e.s.d. in the dihedral angle between two l.s. planes) are estimated using the full covariance matrix. The cell e.s.d.'s are taken into account individually in the estimation of e.s.d.'s in distances, angles and torsion angles; correlations between e.s.d.'s in cell parameters are only used when they are defined by crystal symmetry. An approximate (isotropic) treatment of cell e.s.d.'s is used for estimating e.s.d.'s involving l.s. planes.

### Fractional atomic coordinates and isotropic or equivalent isotropic displacement parameters (Å^2^)

**Table:**

	*x*	*y*	*z*	*U*_iso_*/*U*_eq_	
C1	0.51033 (11)	1.03657 (16)	0.11957 (9)	0.0372 (3)	
C2	0.62740 (13)	1.0134 (2)	0.14228 (11)	0.0488 (3)	
H2	0.6794	1.0968	0.1382	0.059*	
C3	0.66431 (13)	0.8654 (2)	0.17072 (12)	0.0541 (4)	
H3	0.7420	0.8486	0.1863	0.065*	
C4	0.58737 (14)	0.7403 (2)	0.17652 (11)	0.0509 (4)	
H4	0.6148	0.6412	0.1961	0.061*	
C5	0.47145 (13)	0.75842 (17)	0.15415 (10)	0.0442 (3)	
H5	0.4203	0.6739	0.1581	0.053*	
C6	0.43434 (11)	0.90840 (16)	0.12546 (9)	0.0356 (3)	
C7	0.33085 (13)	1.12984 (17)	0.07792 (10)	0.0429 (3)	
C8	0.44359 (12)	1.17348 (17)	0.08955 (9)	0.0412 (3)	
C9	0.22868 (16)	1.2312 (2)	0.05301 (14)	0.0655 (5)	
H9A	0.2527	1.3399	0.0472	0.098*	
H9B	0.1747	1.2233	0.1012	0.098*	
H9C	0.1929	1.1958	−0.0053	0.098*	
C10	0.48781 (17)	1.3321 (2)	0.07368 (12)	0.0559 (4)	
H10	0.4355	1.4109	0.0545	0.067*	
C11	0.13200 (11)	0.92243 (17)	0.19354 (9)	0.0395 (3)	
C12	0.18938 (12)	0.90248 (19)	0.27999 (10)	0.0457 (3)	
H12	0.2640	0.8624	0.2845	0.055*	
C13	0.13415 (16)	0.9431 (2)	0.35933 (11)	0.0569 (4)	
H13	0.1717	0.9313	0.4180	0.068*	
C14	0.02338 (16)	1.0010 (2)	0.35188 (13)	0.0624 (4)	
H14	−0.0138	1.0270	0.4058	0.075*	
C15	−0.03304 (14)	1.0211 (2)	0.26584 (13)	0.0613 (4)	
H15	−0.1078	1.0608	0.2618	0.074*	
C16	0.02108 (12)	0.9824 (2)	0.18524 (11)	0.0516 (4)	
H16	−0.0163	0.9964	0.1267	0.062*	
N1	0.32271 (9)	0.96653 (14)	0.09734 (8)	0.0411 (3)	
O1	0.23308 (10)	0.69878 (14)	0.10309 (9)	0.0575 (3)	
O2	0.13304 (10)	0.91043 (19)	0.01249 (8)	0.0667 (4)	
O3	0.58819 (13)	1.36833 (15)	0.08380 (10)	0.0742 (4)	
S1	0.20015 (3)	0.86138 (5)	0.09314 (2)	0.04468 (12)	

### Atomic displacement parameters (Å^2^)

**Table:**

	*U*^11^	*U*^22^	*U*^33^	*U*^12^	*U*^13^	*U*^23^
C1	0.0448 (6)	0.0379 (7)	0.0289 (6)	−0.0030 (5)	0.0030 (5)	−0.0032 (5)
C2	0.0440 (7)	0.0547 (9)	0.0472 (8)	−0.0074 (6)	−0.0017 (6)	−0.0046 (7)
C3	0.0436 (7)	0.0656 (11)	0.0522 (9)	0.0079 (7)	−0.0060 (6)	−0.0027 (8)
C4	0.0571 (8)	0.0483 (9)	0.0471 (8)	0.0133 (7)	0.0004 (6)	0.0037 (7)
C5	0.0496 (7)	0.0387 (7)	0.0446 (7)	0.0006 (6)	0.0056 (6)	0.0048 (6)
C6	0.0385 (6)	0.0379 (7)	0.0307 (6)	0.0009 (5)	0.0045 (5)	0.0004 (5)
C7	0.0510 (7)	0.0415 (7)	0.0364 (7)	0.0070 (6)	0.0045 (6)	0.0049 (6)
C8	0.0537 (8)	0.0364 (7)	0.0336 (6)	−0.0022 (6)	0.0026 (5)	−0.0003 (5)
C9	0.0608 (10)	0.0607 (11)	0.0750 (12)	0.0181 (8)	0.0052 (9)	0.0158 (9)
C10	0.0760 (11)	0.0397 (8)	0.0511 (9)	−0.0074 (7)	−0.0044 (8)	0.0027 (7)
C11	0.0338 (6)	0.0439 (7)	0.0407 (7)	−0.0018 (5)	0.0020 (5)	0.0007 (6)
C12	0.0411 (7)	0.0510 (8)	0.0445 (8)	0.0027 (6)	−0.0011 (6)	0.0027 (6)
C13	0.0653 (10)	0.0651 (11)	0.0400 (8)	0.0036 (8)	0.0012 (7)	0.0007 (7)
C14	0.0630 (10)	0.0713 (12)	0.0545 (10)	0.0056 (9)	0.0169 (8)	−0.0067 (9)
C15	0.0439 (8)	0.0717 (12)	0.0690 (11)	0.0132 (8)	0.0091 (7)	−0.0060 (9)
C16	0.0385 (7)	0.0648 (10)	0.0508 (8)	0.0050 (6)	−0.0042 (6)	0.0012 (7)
N1	0.0372 (5)	0.0421 (7)	0.0442 (6)	−0.0003 (4)	0.0050 (4)	0.0063 (5)
O1	0.0532 (6)	0.0470 (6)	0.0737 (8)	−0.0101 (5)	0.0156 (5)	−0.0129 (6)
O2	0.0536 (6)	0.1042 (11)	0.0411 (6)	−0.0050 (7)	−0.0075 (5)	−0.0038 (6)
O3	0.0809 (9)	0.0546 (8)	0.0847 (10)	−0.0268 (7)	−0.0179 (7)	0.0078 (7)
S1	0.03861 (17)	0.0548 (2)	0.0407 (2)	−0.00535 (14)	0.00263 (13)	−0.00445 (15)

### Geometric parameters (Å, °)

**Table:**

C1—C2	1.3962 (19)	C9—H9C	0.96
C1—C6	1.3988 (18)	C10—O3	1.207 (2)
C1—C8	1.4404 (19)	C10—H10	0.93
C2—C3	1.370 (2)	C11—C12	1.3835 (19)
C2—H2	0.93	C11—C16	1.3841 (19)
C3—C4	1.386 (2)	C11—S1	1.7552 (14)
C3—H3	0.93	C12—C13	1.378 (2)
C4—C5	1.377 (2)	C12—H12	0.93
C4—H4	0.93	C13—C14	1.376 (2)
C5—C6	1.3871 (19)	C13—H13	0.93
C5—H5	0.93	C14—C15	1.374 (3)
C6—N1	1.4246 (16)	C14—H14	0.93
C7—C8	1.363 (2)	C15—C16	1.383 (2)
C7—N1	1.4046 (19)	C15—H15	0.93
C7—C9	1.490 (2)	C16—H16	0.93
C8—C10	1.451 (2)	N1—S1	1.6753 (12)
C9—H9A	0.96	O1—S1	1.4242 (13)
C9—H9B	0.96	O2—S1	1.4190 (12)
			
C2—C1—C6	119.32 (13)	O3—C10—C8	124.15 (17)
C2—C1—C8	133.15 (13)	O3—C10—H10	117.9
C6—C1—C8	107.53 (12)	C8—C10—H10	117.9
C3—C2—C1	118.80 (14)	C12—C11—C16	121.52 (13)
C3—C2—H2	120.6	C12—C11—S1	118.64 (10)
C1—C2—H2	120.6	C16—C11—S1	119.77 (11)
C2—C3—C4	120.88 (14)	C13—C12—C11	118.90 (14)
C2—C3—H3	119.6	C13—C12—H12	120.6
C4—C3—H3	119.6	C11—C12—H12	120.6
C5—C4—C3	121.96 (15)	C14—C13—C12	120.02 (15)
C5—C4—H4	119.0	C14—C13—H13	120.0
C3—C4—H4	119.0	C12—C13—H13	120.0
C4—C5—C6	117.02 (14)	C15—C14—C13	120.82 (15)
C4—C5—H5	121.5	C15—C14—H14	119.6
C6—C5—H5	121.5	C13—C14—H14	119.6
C5—C6—C1	122.02 (12)	C14—C15—C16	120.14 (15)
C5—C6—N1	131.25 (12)	C14—C15—H15	119.9
C1—C6—N1	106.74 (12)	C16—C15—H15	119.9
C8—C7—N1	108.28 (12)	C15—C16—C11	118.59 (14)
C8—C7—C9	128.68 (14)	C15—C16—H16	120.7
N1—C7—C9	123.01 (14)	C11—C16—H16	120.7
C7—C8—C1	108.71 (12)	C7—N1—C6	108.71 (11)
C7—C8—C10	125.09 (14)	C7—N1—S1	125.08 (10)
C1—C8—C10	126.20 (14)	C6—N1—S1	126.14 (10)
C7—C9—H9A	109.5	O2—S1—O1	119.54 (8)
C7—C9—H9B	109.5	O2—S1—N1	107.77 (7)
H9A—C9—H9B	109.5	O1—S1—N1	106.19 (6)
C7—C9—H9C	109.5	O2—S1—C11	109.16 (7)
H9A—C9—H9C	109.5	O1—S1—C11	109.24 (7)
H9B—C9—H9C	109.5	N1—S1—C11	103.76 (6)
			
C6—C1—C2—C3	−0.7 (2)	C13—C14—C15—C16	0.2 (3)
C8—C1—C2—C3	178.78 (15)	C14—C15—C16—C11	0.5 (3)
C1—C2—C3—C4	0.3 (2)	C12—C11—C16—C15	−0.7 (2)
C2—C3—C4—C5	0.1 (2)	S1—C11—C16—C15	176.34 (13)
C3—C4—C5—C6	−0.1 (2)	C8—C7—N1—C6	2.23 (15)
C4—C5—C6—C1	−0.2 (2)	C9—C7—N1—C6	−175.57 (14)
C4—C5—C6—N1	179.63 (14)	C8—C7—N1—S1	179.33 (10)
C2—C1—C6—C5	0.6 (2)	C9—C7—N1—S1	1.5 (2)
C8—C1—C6—C5	−178.94 (12)	C5—C6—N1—C7	178.03 (14)
C2—C1—C6—N1	−179.26 (12)	C1—C6—N1—C7	−2.08 (14)
C8—C1—C6—N1	1.17 (14)	C5—C6—N1—S1	1.0 (2)
N1—C7—C8—C1	−1.48 (16)	C1—C6—N1—S1	−179.15 (9)
C9—C7—C8—C1	176.15 (15)	C7—N1—S1—O2	41.89 (14)
N1—C7—C8—C10	178.30 (13)	C6—N1—S1—O2	−141.51 (11)
C9—C7—C8—C10	−4.1 (3)	C7—N1—S1—O1	171.10 (11)
C2—C1—C8—C7	−179.31 (15)	C6—N1—S1—O1	−12.30 (13)
C6—C1—C8—C7	0.18 (15)	C7—N1—S1—C11	−73.79 (13)
C2—C1—C8—C10	0.9 (2)	C6—N1—S1—C11	102.82 (12)
C6—C1—C8—C10	−179.60 (13)	C12—C11—S1—O2	−172.81 (12)
C7—C8—C10—O3	−179.51 (16)	C16—C11—S1—O2	10.03 (16)
C1—C8—C10—O3	0.2 (3)	C12—C11—S1—O1	54.80 (13)
C16—C11—C12—C13	0.2 (2)	C16—C11—S1—O1	−122.36 (13)
S1—C11—C12—C13	−176.90 (13)	C12—C11—S1—N1	−58.13 (13)
C11—C12—C13—C14	0.6 (3)	C16—C11—S1—N1	124.72 (13)
C12—C13—C14—C15	−0.8 (3)		

### Hydrogen-bond geometry (Å, °)

**Table:**

*D*—H···*A*	*D*—H	H···*A*	*D*···*A*	*D*—H···*A*
C5—H5···O1	0.93	2.28	2.8723 (19)	121
C12—H12···O3^i^	0.93	2.48	3.1664 (19)	131
C16—H16···O2^ii^	0.93	2.48	3.388 (2)	167

Symmetry codes: (i) −*x*+1, *y*−1/2, −*z*+1/2; (ii) −*x*, −*y*+2, −*z*.

## References

[bb1] Andreani, A., Granaiola, M., Leoni, A., Locatelli, A., Morigi, R., Rambaldi, M., Giorgi, G., Salvini, L. & Garaliene, V. (2001). *Anti-Cancer Drug Des.* **16**, 167–174. 11962514

[bb2] Bernstein, J., Davis, R. E., Shimoni, L. & Chang, N.-L. (1995). *Angew. Chem. Int. Ed. Engl.* **34**, 1555–1573.

[bb3] Bruker (2004). *APEX2* and *SAINT* Bruker AXS Inc., Madison, Wisconsin, USA.

[bb4] Chai, H., Zhao, C. & Gong, P. (2006). *Bioorg. Med. Chem.* **14**, 911–917. 10.1016/j.bmc.2005.08.041 16183290

[bb5] Chakkaravarthi, G., Dhayalan, V., Mohanakrishnan, A. K. & Manivannan, V. (2008). *Acta Cryst.* E**64**, o749. 10.1107/S1600536808007678 PMC2960947 21202139

[bb6] Chakkaravarthi, G., Ramesh, N., Mohanakrishnan, A. K. & Manivannan, V. (2007). *Acta Cryst.* E**63**, o3564.

[bb7] Ramathilagam, C., Saravanan, V., Mohanakrishnan, A. K., Chakkaravarthi, G., Umarani, P. R. & Manivannan, V. (2011). *Acta Cryst.* E**67**, o632. 10.1107/S1600536811004685 PMC3051985 21522386

[bb8] Sheldrick, G. M. (1996). *SADABS* University of Göttingen, Germany.

[bb9] Sheldrick, G. M. (2008). *Acta Cryst.* A**64**, 112–122. 10.1107/S0108767307043930 18156677

[bb10] Singh, U. P., Sarma, B. K., Mishra, P. K. & Ray, A. B. (2000). *Folia Microbiol. (Prague)*, **45**, 173–176. 10.1007/BF02817419 11271828

[bb11] Spek, A. L. (2009). *Acta Cryst.* D**65**, 148–155. 10.1107/S090744490804362X PMC2631630 19171970

